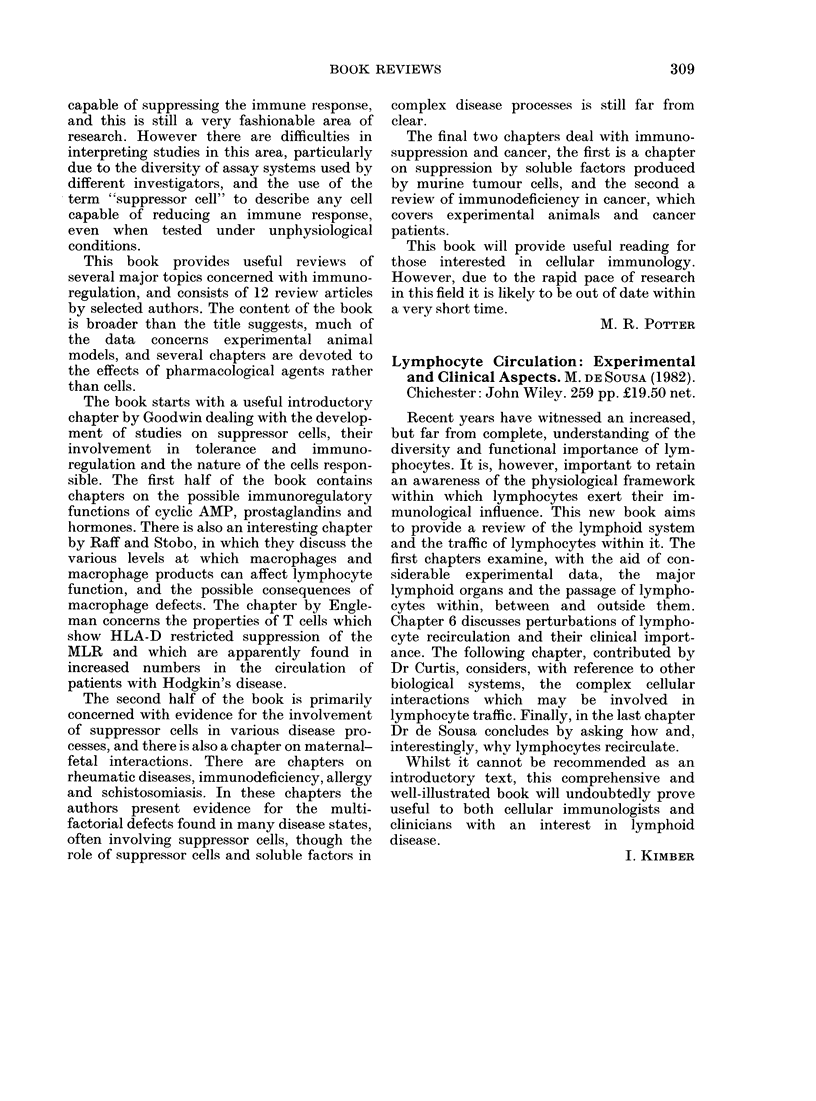# Lymphocyte Circulation: Experimental and Clinical Aspects

**Published:** 1982-08

**Authors:** I. Kimber


**Lymphocyte Circulation: Experimental and Clinical Aspects.** M. DE SOUSA (1982). Chichester: John Wiley. 259 pp. £19.50 net.

Recent years have witnessed an increased, but far from complete, understanding of the diversity and functional importance of lymphocytes. It is, however, important to retain an awareness of the physiological framework within which lymphocytes exert their immunological influence. This new book aims to provide a review of the lymphoid system and the traffic of lymphocytes within it. The first chapters examine, with the aid of considerable experimental data, the major lymphoid organs and the passage of lymphocytes within, between and outside them. Chapter 6 discusses perturbations of lymphocyte recirculation and their clinical importance. The following chapter, contributed by Dr Curtis, considers, with reference to other biological systems, the complex cellular interactions which may be involved in lymphocyte traffic. Finally, in the last chapter Dr de Sousa concludes by asking how and, interestingly, why lymphocytes recirculate.

Whilst it cannot be recommended as an introductory text, this comprehensive and well-illustrated book will undoubtedly prove useful to both cellular immunologists and clinicians with an interest in lymphoid disease.

I. KIMBER